# *In vitro* retention capacity of two overdenture attachment systems: Locator® and Equator®

**DOI:** 10.4317/jced.54834

**Published:** 2018-07-01

**Authors:** Nieves Mínguez-Tomás, Jorge Alonso-Pérez-Barquero, Lucía Fernández-Estevan, Ángel Vicente-Escuder, Eduardo J. Selva-Otaolaurruchi

**Affiliations:** 1 DDS, MSc, Department of Prosthodontics and Occlusion, Stomatology Department, Faculty of Medicine and Dentistry, University of Valencia, Valencia, Spain; 2 DDS, MSc, PhD Department of Prosthodontics and Occlusion, Stomatology Department, Faculty of Medicine and Dentistry, University of Valencia, Valencia, Spain; 3 B.Sc, MSc, Institute of Materials Technology. Laboratory of dynamic tests and fracture. Polytechnic University of Valencia, Spain; 4 DMD, PhD, Department of Prosthodontics and Occlusion, Stomatology Department, Faculty of Medicine and Dentistry, University of Valencia, Valencia, Spain

## Abstract

**Background:**

It is necessary to know the *in vitro* behavior of different attachment systems to be used clinically. The evolution of retention capacity over 10 years (14,600 insertion/de-insertion cycles) was determined *in vitro*, evaluating two overdenture attachment systems (Locator® and OT Equator®).

**Material and Methods:**

The study used an implant replica compatible with the abutments of both systems. 10 Locator® and 10 OT Equator® attachments were screwed to the abutments. Nylon inserts were attached and tested, subjecting them to 14,600 insertion and de-insertion cycles (representing 10 years functional life) in axial direction. The universal test machine crosshead speed was 50 mm/min with a de-insertion range of 2 mm.

**Results:**

The initial retention of Locator® was 17.02 N and of Equator® 16.36 N. After 14,600 cycles, Locator® suffered a mean loss of retention of 50.89%, while Equator® lost 69.28%. Both systems showed retention increases up to the first 1,000 cycles, which decreased thereafter up to 14.600 cycles. Statistically significant differences between the systems were found after 7,500 cycles.

**Conclusions:**

Both systems presented acceptable retention capacities after 14,600 cycles. Significant differences in retention force between the systems evolved after 7,500 cycles (5 years *in vitro* use). These results should be treated with caution and should be verified clinically.

** Key words:**Denture, mandibular prosthesis implantation, attachment, dental implant-abutment connection, denture retention.

## Introduction

For many years, the quality of life of edentulous patients rehabilitated with complete removable prostheses has been compromised by the overdenture’s lack of stability and retention on the alveolar process, particularly in the mandibular arch (1,2). In 2002, the McGill Consensus statement established a first-choice standard of care for treating edentulous patients: overdentures (OD) supported by two osseointegrated implants placed in canine position, and retained by an attachment system (3-15). The general acceptance of this type of treatment has led to the advent of a wide range of anchorage systems that are constantly evolving in design to meet the needs of both patients and clinicians (16,17).

The Locator® system (Zest Anchor, Escondido, CA) has been widely researched in vitro due to its reduced size, its retention capacity over time, and its better tolerance of angulation between implants than provided by other systems (7,9,10,12,18-22). *In vivo* studies also vouch for the system 11,21

The OT Equator® system (Rhein 83, Bologna, IT) was launched in 2007, offering an attachment of reduced size that is useful when prosthetic space is compromised. But the system has not been widely investigated and there are few references in the literature that vouch for its clinical adequacy. As retention is one of the most important characteristics as far as OD-wearing patients are concerned, the present study set out to evaluate the OT Equator® system’s retention capacity in comparison with the more widely researched Locator system (6,9,10,18). The retention capacity of both systems was evaluated before, during and after 14,600 cycles of insertion and de-insertion; this number of cycles is equivalent to 10 years functional life in the mouth, removing the prosthesis four times a day for cleaning and disinfection. 18 So the objective of this assay was to compare the evolution of the retention capacity of two similar overdenture attachment systems retention over 14,600 insertion and de-insertion cycles. The assay’s null hypothesis was that the OT Equator has a similar retention capacity to the Locator system.

## Material and Methods

This *in vitro* study evaluated two stud-type attachments of similar characteristics: the Locator® (Zest Anchors Inc, Escondido, CA, USA) and the OT Equator® (Rhein83, Bologna, Italy).

**Figure 1:**
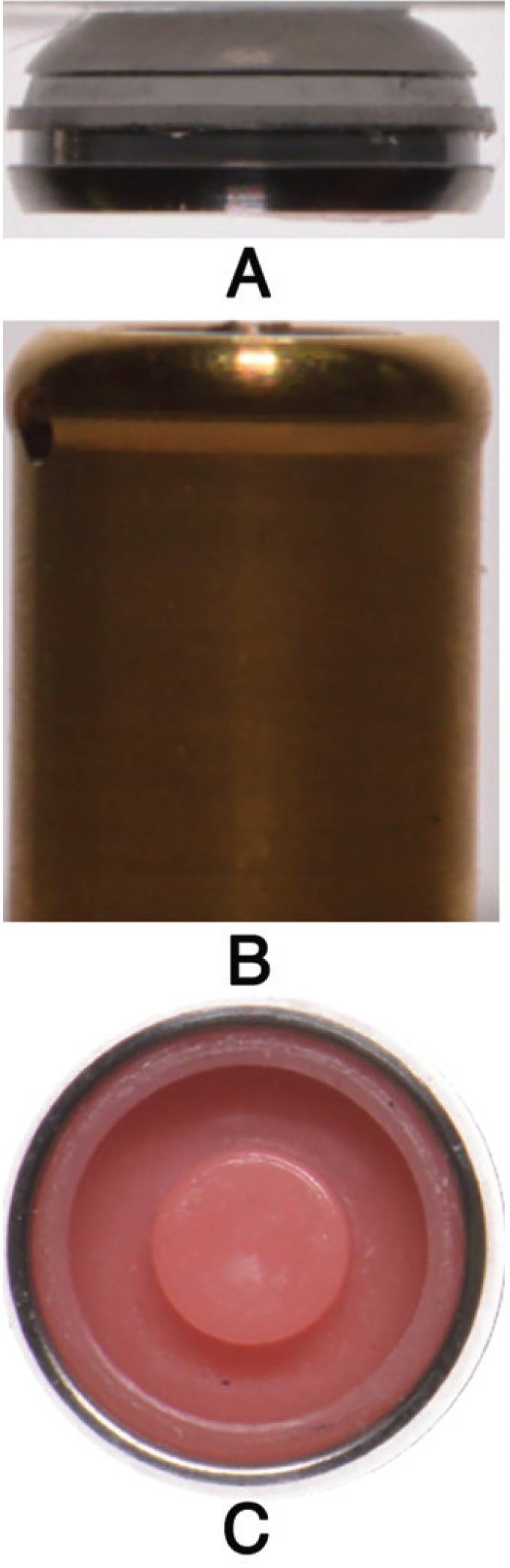
A, Locator Abutment (male part) B, Locator housing with pink nylon insert (female part) C, Locator housing (female part). Lateral view.

The Locator attachment is self-aligning and offers double retention consisting of two parts: the male part consists of a titanium abutment with a hard coating of titanium nitrite which screws to the implant (Fig. 1A); the female part is a titanium cap which is inserted into the acrylic overdenture and houses changeable nylon retention inserts (Fig. 1C). There are six different inserts available with different retentive strengths that are color-coded and vary from 1.5 to 5.3 lbs. (6.66 N to 22.26 N) (8,21).

**Figure 2:**
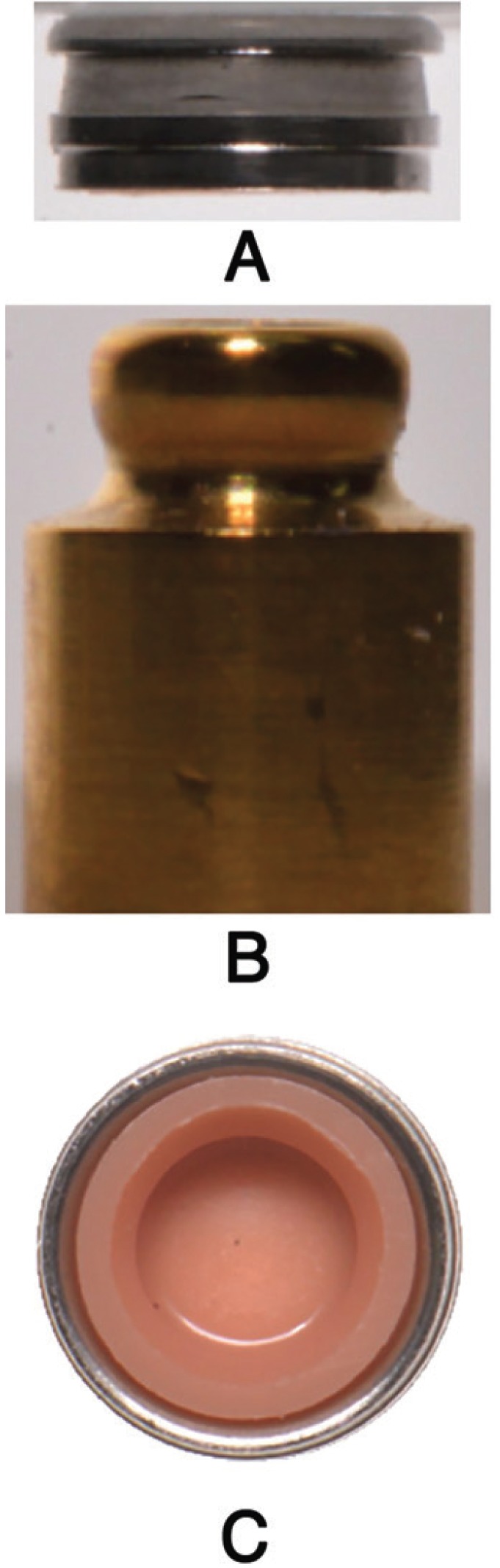
A, Equator Abutment (male part), B, Equator housing with pink nylon insert (female part), C, Equator housing (female part). Lateral view.

The OT Equator attachment has similar characteristics, a titanium male part (Fig. 2A); with titanium nitrite coating and a semispherical shape reminiscent of ball attachments that supports a stainless steel retentive cap housing nylon retentive inserts available with four levels of retention ranging from 1.3 to 5.9 lbs. (5.87 N to 26.47 N), also color-coded (like the Locator system) (Fig. 1).

The nylon inserts selected for the present study were of pink color for both systems and with similar retention strengths (Locator: 13.33 N; Equator: 11.76 N).

Two groups of 10 complexes per system were created, each complex consisting of an abutment, a housing, and a nylon insert.

The first study group (Locator), included 10 Locator abutments with 3mm tissue cuff height, 10 titanium retentive housings of 5.45 mm diameter and 2.35 mm height, and 13.33 N pink nylon retention inserts. The second group (Equator) consisted of 10 OT Equator abutments of 3 mm tissue cuff height, with 10 stainless steel caps of 4.4 mm diameter and 2.1 mm height, and 11.76 N pink nylon inserts.

Each abutment was screwed to a replica implant with a 4.1 mm diameter platform and Parangon® internal connection (Zimmer, Warsaw, Ind), compatible with both systems.

The implant replicas were embedded in a plastic cylinder with Exakto-Form® resin (Bredent, Senden, Germany). A device was designed to ensure perfect alignment at 0o to the implant axis. Then the nylon insert was placed in its cap and this onto the abutment, checking that alignment of the abutment-insert-cap complex was totally axial.

When the complex was perfectly aligned, the housing was fixed with Araldite® (Huntsman, Tx) to an aluminum container that was then attached to the test machine’s upper crosshead.

The test machine was programmed to perform 14,600 insertion/de-insertion cycles for each of the 10 specimens in each group. The cycle consisted of an upwards movement of 2 mm at a crosshead speed of 50 mm/min, and a downwards movement of the same characteristics (23-25). The study used an Instron® 8874 universal test machine (Instron, Ma), which programmed upwards and downwards movements, frequency, and registered retention strength data for each and every one of the 14,600 cycles (18). A 100 kN load cell was used, together with Instron® Wave Maker Editor 7.0.0 software. All results were registered in Newtons.

Retention force data were registered for each and every cycle (1 to 14.600). For statistical analysis, the means of twenty values at cycles 0, 100, 500, 1000, 2000, 3000, 4000, 5000, 7500, 10.000 and 14.600 were calculated.

Statistical analysis consisted of calculating descriptive statistics of the retention force variable (mean, standard deviation, range and median) by group (attachment system). Inferential analysis consisted of estimating a Brunner-Langer non-parametric model for correlated data. An ANOVA-type statistic (ATS) was calculated to evaluate principle effects and interaction.

Statistical analysis was performed using specialized software (SPPS 15.0, Chicago, Illinois, USA). The non-parametric Mann-Whitney test was used to evaluate data distribution homogeneity of strength values in both groups at a determined moment in the cycle test. Percentages of loss of strength from start to finish were compared analogically.

The significance level was set at 5% (*P*<0.05). Statistical tests reached a power of 0.40 in order to detect a difference of 2 N as significant (compatible with an effect size of 0.8) assuming a confidence level of 95%.

## Results

 Table 1 shows descriptive statistics for changing retention strength in the two study groups over the cycle sequence described above.

**Table 1:**
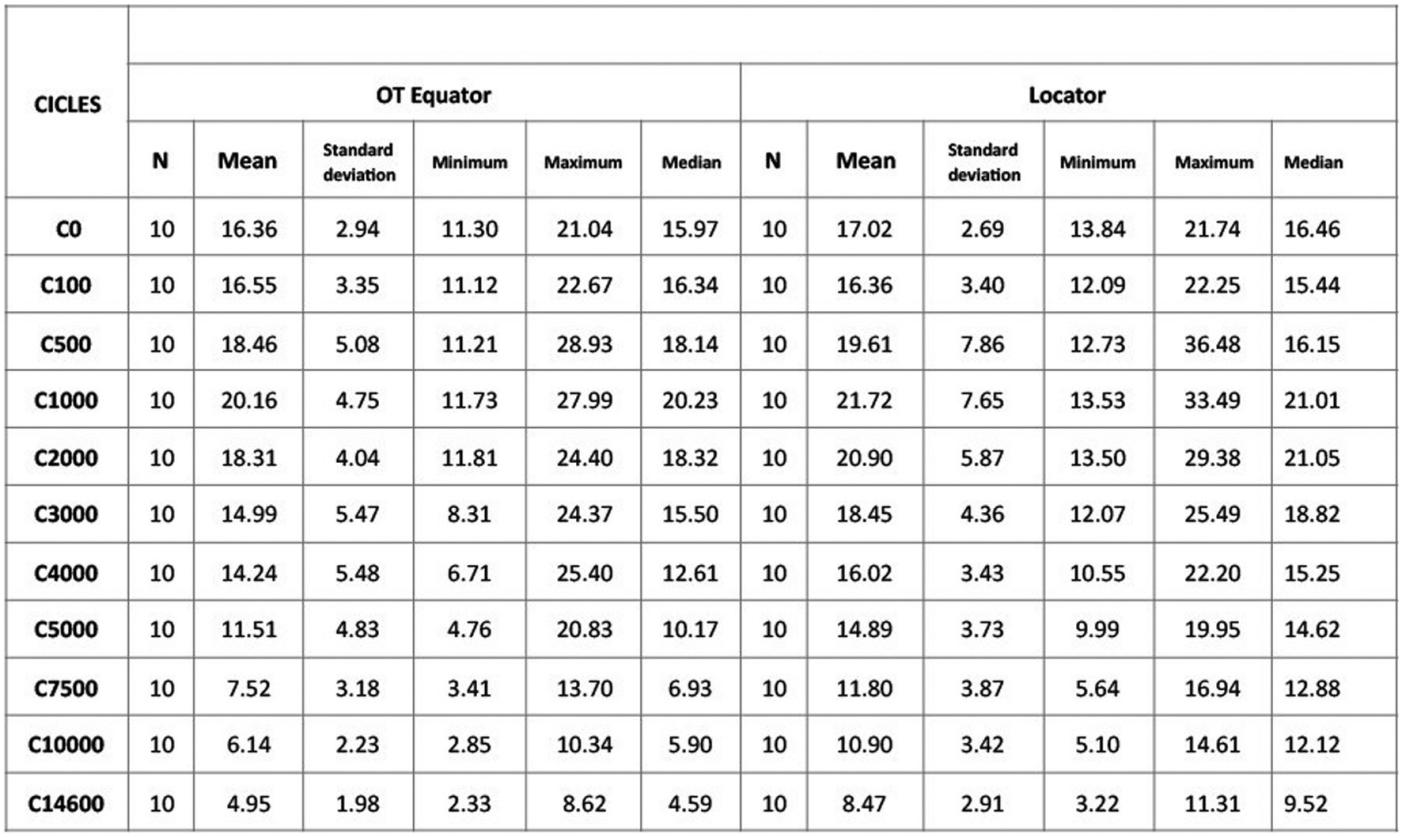
Evolution of retention strength in both groups according to number of insertion/de-insertion cycles.

With the Locator system, mean retention fell from 17.02 ± 2.69 N at baseline to 8.47 ± 2.91 N after 14,600 cycles. With the OT Equator system, retention fell from 16.36 ± 2.94 N to 4.95 ± 1.98 N at the end of the cycle sequence.

**Figure 3:**
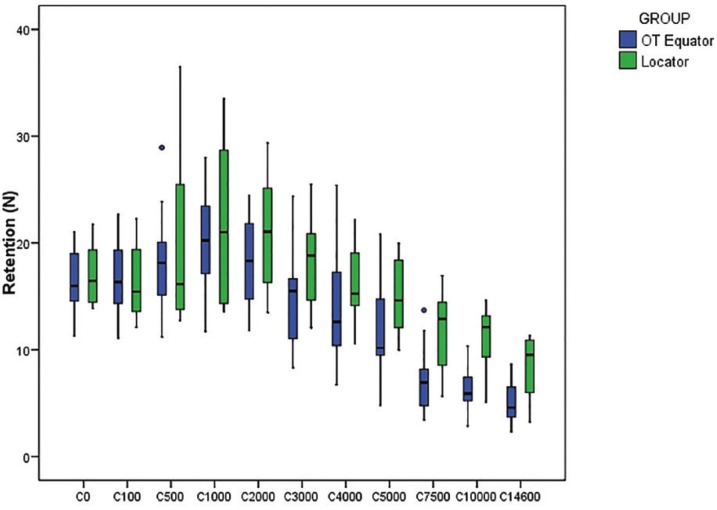
Box plot shows the distribution of retention values obtained by the two groups.

The box plot shows the distribution of retention values obtained by the two groups (Fig. 3).

Both systems underwent an increase in retention strength over the first 1,000 cycles (representing 8 months functional life), the Locator system reaching 21.72 ± 7.65 N and OT Equator 20.16 ± 4.75 N. Until the 1,000-cycle point, both systems presented similar retention values although greater variation between samples was observed in the Locator group. From the 1,000th to the 2,000th cycle, median values were higher in the Locator group than the Equator group, with the greatest difference occurring around the 7,500th cycle mark. Variation between samples was also greater in the Locator group after the 7,500th cycle.

The Brunner-Langer model was applied and an ATS estimated to evaluate different effects: group, number of cycles, and interaction between variables. No statistically significant differences were found between retention values across the whole cycle sequence (*P*=.115). There was insufficient evidence to confirm significant differences between retention curves (*P*=.210). However, a statistically significant difference was confirmed between different test cycles (*P*<.001).

The method employed contemplated the existence of intra-model correlations.

There were differences in the baseline retention values between the 10 samples in each group. These differences continued throughout the sequence of cycles, so that each individual sample obtained disparate values. So clearly, there was a degree of dispersion in the results, with retention capacity varying between one sample and another. The Mann-Whitney test confirmed that differences in retention grew as fatigue testing advanced ( Table 2). But it was only after the 7,500th cycle until the last cycle that the higher retention values registered in the Locator group reached statistically significant difference in comparison with the Equator group.

**Table 2:**
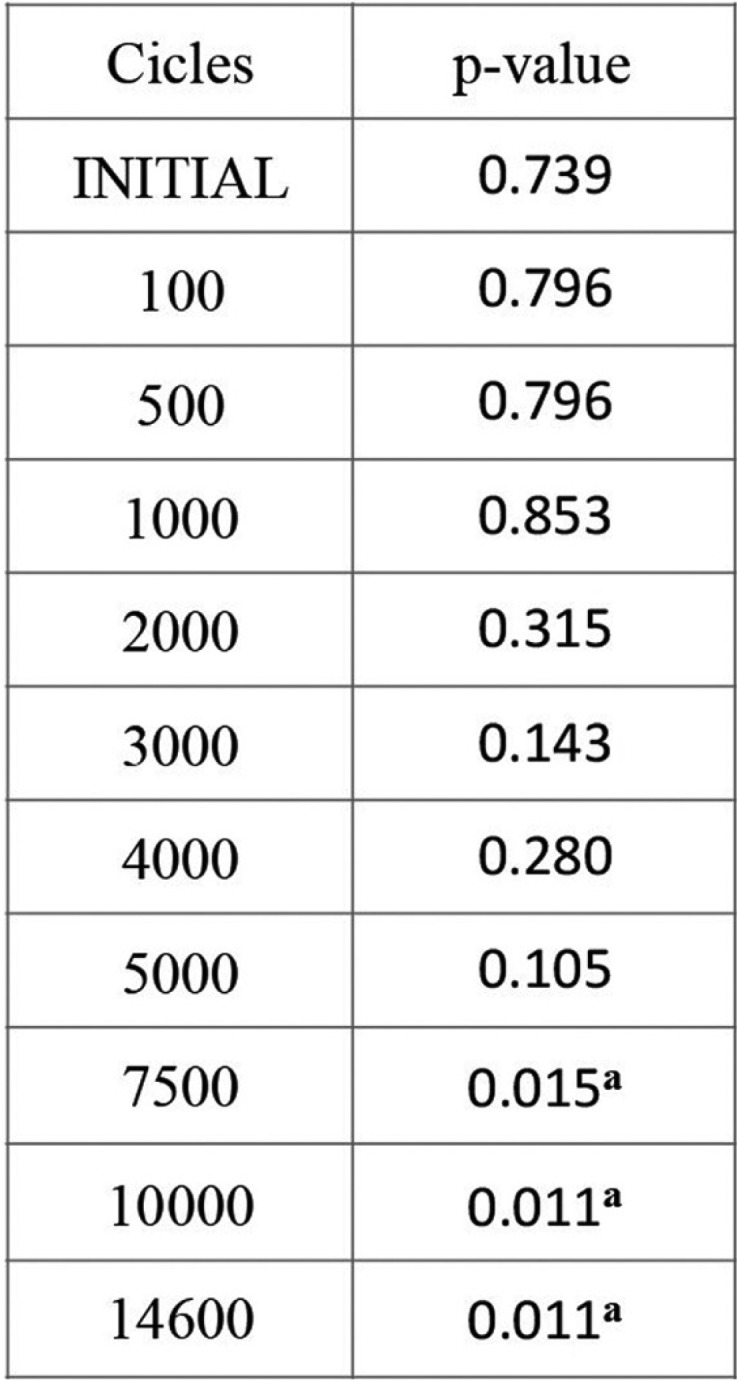
Mann Whitney Test Results. ap<.05

In the Locator group, the mean percentage of retention loss was 48.8 ± 18.8% and in the Equator group 68.1 ± 17.8%. The Mann Whitney test applied to compare initial and final retention identified significant differences (*P*=.029) between median values, implying less retention loss with the Locator system throughout the fatigue testing cycles.

## Discussion

The present study compared the evolution of the retention capacity of two overdenture attachment systems (Locator® and OT Equator®) subjected to fatigue testing consisting of 14,600 insertion/de-insertion cycles. This number represents the everyday usage of the prosthesis over a ten-year period, estimating that the wearer would remove and reinsert the overdenture four times a day (18). Although some authors such as Besimo (4) and Gamborena (5) have used smaller numbers of cycles in similar studies, the present authors agree with Rutkunas (9) that 14,600 cycles offer more accurate information about the wear to attachments and their loss of retention.

The study used a single attachment per sample, which differs from other researchers who have used experimental models consisting of a pair of attachments or by replicating entire overdentures to test their retention (10,15,18). In the present study, the one-piece attachments were positioned axially in order to study the behavior of each complex without the influence of factors such as non-parallelism between attachments (which other researchers have investigated) (6,9,20). In the same way, the force exerted to insert and de-insert was also applied axially, as the objective was to determine the evolution of retention capacity without the influence of factors such as divergence.

Al-Ghafli (18) suggested that the number and position of implants, the type of material used to fabricate the attachments, prosthetic design, and diverse forces of different magnitudes in different directions all represent factors that may influence retention loss. This wide variety of factors makes it difficult to reproduce real clinical conditions *in vitro*. Rutkunas (9), performed an *in vitro* study observing signs of wear under scanning electron microscopy after one-piece attachments were subjected to 15,000 cycles with vertical traction; Rutkunas explains wear as the result of the friction produced between the male and female retention elements. Some authors (9,21) have proposed that some attachment parts need to be changed more often than is reflected in *in vitro* studies. Furthermore, it might be that the wear to the nylon inserts is not only due to the action of placing and removing the overdenture, but to the simple usage of the prosthesis in the normal daily routine.

Many other researchers (9-12,23) have used the same crosshead speed as the present study: 50 mm/min. In 1983, Sarnat (23) proposed this speed as close to the speed of the movement of real overdenture removal from its retention elements when vertical force is applied. Since then, many others (15,24,25) have adopted this speed to test retention based on Sarnat’s proposal but there is no clear evidence that this is the actual speed of OD removal. When the present study was designed, it was thought that matching removal speed to most previous studies would facilitate comparison between results, and in any case this speed is sufficiently slow to avoid damaging the nylon polymer.

Adequate retention is associated with improved patient satisfaction (2,3) and so increased quality of life. But there is little consensus in the literature as to the minimum retention required to maintain an acceptable level of satisfaction among wearers of ODs. Various values have been proposed as adequate. Caldwell (26) Trakas (13) and Setz (17) coincide in that the minimum retention required for mandibular ODs with the use of one-piece attachments varies between 10 N and 20 N. Pigozzo (16) suggests that retention of 5-7 N is enough to keep an OD stable. When considering the minimum retention capacity of a single one-piece attachment, various authors propose values ranging from 3 to 8 N (1,2). The present study obtained final retention values, after 14,600 cycles, of 8.47 ± 2.91 N for the Locator system and 4.95 ± 1.98 N for the Equator system, which are within the limits described as acceptable for maintaining OD stability.

The retention capacity of the two systems studied did not coincide with the values announced by their manufacturers. In the case of the Locator system with pink nylon inserts, the manufacturer reports a retention force of 13.33 N, while at cycle 0, the study obtained a value of 17.02 ± 2.55 N and it was not until the 10.000th cycle (12,12 N) that values were obtained close to those claimed by the manufacturer. As for the Equator system with pink nylon inserts, the manufacturer claims a retention capacity of 11.76N, while the study obtained a value of 15.97 ± 2.94 N at baseline, and values close to those reported by the manufacturer were not observed until the 5,000th cycle.

There is a remarkable disparity of results between different evaluations of the baseline retention capacity of the Locator system with pink inserts. Testing single attachments, Rutkunas (12) obtained baseline retention of 10.6 ± 1.24 N, Alsabeeha 6 obtained 9.40 ± 0.74 N, and Wolf 20 13.25 ± 6.6 N. Testing two attachments retaining a replica OD, Scherer (7) obtained 26.61 N, while Chung (10) reported a retention strength of only 12.33 ± 1.28 N. Variations in baseline retention has also been obtained within single groups in the same study, which Wolf (20) attributes to tolerance in the manufacture of the retention elements.

In the present study, the Locator and Equator systems showed similar characteristics at baseline with a mean retention capacity of 17.02 ± 2.69 N and 16.36 ± 2.94 N respectively. Thereafter, their retention capacity increased up to the 1,000th cycle reaching a mean value of 21.72 ± 7.65 N, a 27.61% increase from the baseline value for the Locator system, while the Equator system increased to 20.16 ± 4.75 N, a 23.22% increase from baseline. Other authors have observed similar behavior (4,23,26) Al-Ghafli (18) attributed this effect to increased surface roughness on the nylon insert resulting from early wear.

After this initial increase, both systems then started to lose retention progressively, but their behavior continued to show similar patterns, without statistically significant differences until the 7,500th cycle. At that point, the Locator system maintained 69.33% of its baseline retention, while OT Equator obtained mean retention of 7.52 ± 3.18 N, 45.96% of baseline retention. Thereafter, these significant differences continued until the study’s final 14,600th cycle, when the Locator system obtained mean retention of 8.47 ± 2.91 N (49.76% of baseline retention), while the OT Equator obtained a mean of 4.95 ± 1.98 N (30.26%). This differs from the study by Rutkunas, 9 who investigated single pink insert Locator attachments, obtaining an initial mean value of 15.20 ± 6.9 N. From baseline, retention underwent a considerable loss of retention until the 750th cycle point when mean retention was 7.5 N, after which it underwent a continuous increase until the 15000th cycle, when it obtained a mean value of 11.95 ± 3.5 N, which represents a 78.6% loss of baseline retention.

Despite the statistically significant differences found after the 7,500th cycle point, both systems nevertheless fulfilled the minimum retention requirements put forward by other authors during this simulation of 10 years functional life.

## Conclusions

Within the limitations of this *in vitro* laboratory study, the following conclusions may be drawn.

- Both the Locator and the OT Equator systems maintain clinically acceptable retention after 10 years usage.

- Retention increases from baseline values until around the 1000-cycle mark (representing 8 months functional life).

- Retention values were similar for the two systems until the 7,500th cycle (5 years).

- After the 7,500-cycle point, statistically significant differences in retention develop between the two systems with OT Equator undergoing a greater loss of retention than Locator.

- More detailed *in vitro* studies are required that better reproduce clinical situations, as well as randomized clinical trials to compare patient satisfaction with different attachment systems.
